# Metformin as a novel organic foliar bio-stimulant to enhance peanut (*Arachis hypogaea* L.) growth and yield under drought stress conditions

**DOI:** 10.1186/s12870-025-06925-9

**Published:** 2025-07-17

**Authors:** Marwa A. Qotb, Ismail A. Abdelhamid, Nader R. Habashy, Abdel Rahman Mohammad Al Tawaha, Abdel Razzaq Al-Tawaha, Arun Karnwal, Tabarak Malik

**Affiliations:** 1 Soils, Water and Environment Research Institute, Agriculture Research Center, Giza, Egypt; 2 Department of Chemistry, Faculty of Science, Cairo University, Giza, 12613 Egypt; 3 Department of Biological Sciences, Al Hussein Bin Talal University, PO Box 20, Maan, Jordan; 4 National Agricultural Research Center (NARC), Baq’a, 19381 Jordan; 5 Department of Microbiology, Graphic Era (Deemed to Be University), Dehradun, Uttarakhand 248009 India; 6 Department of Biomedical Sciences, Institute of Health, Jimma University, Jimma, Ethiopia; 7 Division of Research & Development, Lovely Professional University, Punjab 144401 Phagwara, India

**Keywords:** Abiotic stress resilience, Foliar application efficiency, Nutrient assimilation, Crop productivity, Metformin

## Abstract

**Background:**

Drought stress significantly affects peanut (*Arachis hypogaea* L.) growth and yield, necessitating strategies to enhance crop resilience. This study evaluates the impact of foliar-applied Metformin, gibberellic acid (GA₃), and indole-3-acetic acid (IAA) at concentrations of 5.0, 7.5, and 10.0 mg L⁻^1^ under different irrigation regimes (100%, 80%, and 60% of the recommended irrigation rate).

**Methods:**

A two-year field experiment was conducted under arid conditions to assess the effects of these treatments on plant growth, yield, photosynthetic pigments, nutrient uptake, and water use efficiency (WUE). Peanut plants were exposed to three irrigation levels (100%, 80%, and 60%), and foliar treatments were applied at specific growth stages. Photosynthetic parameters, including chlorophyll and carotenoid content, were measured alongside growth and yield attributes to determine treatment efficacy.

**Results:**

The application of Metformin at 7.5 mg L⁻^1^ under 80% irrigation significantly improved plant height (76.9 cm), branch number (17.7 per plant), fresh weight (2928.5 kg acre⁻^1^), dry biomass (329.1 kg acre⁻^1^), and total seed yield (1593.9 kg acre⁻^1^) compared to other treatments. Additionally, water use efficiency (WUE) increased by 50.8% in plants treated with Metformin at 7.5 mg L⁻^1^ under 80% irrigation compared to untreated plants. The highest chlorophyll content (1.27 mg g⁻^1^ FW) and carotenoid levels (2.87 mg g⁻^1^ FW) were observed with Metformin at 7.5 mg L⁻^1^ under 100% irrigation, indicating improved photosynthetic performance.

**Conclusion:**

Foliar application of Metformin at 7.5 mg L⁻^1^ under 80% irrigation effectively enhances peanut growth, yield, and WUE, providing a sustainable strategy to mitigate drought stress effects. This treatment balances crop productivity and water conservation, making it a viable approach for peanut cultivation in water-limited environments.

## Highlights

- Foliar application of Metformin at 7.5 mg L⁻¹ significantly improves peanut growth, yield, and water use efficiency under drought stress.

- Peanut plants treated with Metformin exhibit increased plant height, branch number, fresh and dry biomass, and seed yield.

- The combination of Metformin at 7.5 mg L⁻¹ with 80% irrigation optimally enhances yield while conserving water resources.

- Metformin application increases chlorophyll and carotenoid content, supporting improved photosynthesis and plant vigor.

- The study demonstrates that Metformin can serve as an effective, eco-friendly bio-stimulant for peanut cultivation in water-scarce regions.

## Background

Peanuts (*Arachis hypogea* L.) are among the most widely cultivated oilseeds globally [73, 80]. Originating in South America, peanuts have since spread worldwide and are now recognized as one of the eight most common food allergens [15, 32]. They are grown extensively across many countries, with global production surpassing 48 million tons in 2019 [65]. Nutritionally, peanuts are dense in proteins, oleic acid, antioxidants, essential minerals, and bioactive compounds, including phytosterols, polyphenols, phenolic compounds, stilbenes, lignans, isoflavonoids, and B vitamins [3, 6, 36, 53]. They are also an abundant source of both water- and oil-soluble micronutrients.

Climate change has significantly affected food security in recent years by reducing soil water availability, particularly in vulnerable regions [17, 69]. This decrease in soil water increases stress on limited water resources, notably impacting agricultural productivity in the Mediterranean and Africa [55]. For peanuts, water stress has been shown to hinder crop yields by reducing seed emergence rates, restricting leaf and kernel size, and resulting in weaker plants and heavily wilted fruits [42, 78, 83]. This stress diminishes moisture levels in peanut pods, negatively affecting the seeds'physiological activity and leading to lower seed yield and quality [5, 7, 9, 10, 24, 61, 70, 81].

Egypt faces considerable water limitations, with its primary water supply regulated by the Nile and Lake Nasser's water balance, controlled by releases through the Aswan High Dam [57]. Consequently, water resources are increasingly scarce, leading to soil water stress that challenges crop growth and yield, particularly in peanut production. These water constraints significantly impact peanuts’ growth, development, and yield, limiting yield and quality improvement. Effective irrigation management is thus essential to sustain peanut production in Egypt, conserve water resources, and enhance water use efficiency to support sustainable agriculture. Drip irrigation offers an eco-friendly and water-efficient solution for peanut cultivation, helping address water scarcity. The efficacy of drip irrigation, however, is influenced by soil type and water availability,in this study's experimental site, sandy soil presents unique challenges, such as surface water accumulation and restricted root water uptake, which hinder potential yield improvements, [38, 71]. Increasing water retention capacity in sandy soils is vital for sustaining long-term growth in the peanut sector. An efficient irrigation regime is crucial to address water scarcity while optimizing water distribution for agricultural purposes.

Beyond optimizing irrigation, applying plant growth regulators (PGRs) can help mitigate soil water stress in peanut cultivation. Recent studies have investigated novel pharmaceutical compounds such as PGRs to enhance peanut growth and yield, with Metformin emerging as a promising candidate. Derived from the French lilac (*Galega officinalis*), Metformin is a water-soluble biguanide derivative (1,1-dimethyl biguanide hydrochloride) that, due to its ionic properties, is readily absorbed by roots and translocated primarily to the shoots [22, 58]. While studies on the uptake and movement of pharmaceuticals like Metformin in legume crops are limited, emerging research suggests that Metformin, once metabolized within plants, may resemble nitrogenous compounds like galegine and arginine, potentially benefiting crop growth [22].

In addition to Metformin, two classical PGRs—gibberellic acid (GA₃) and indole-3-acetic acid (IAA)—have been widely studied for their roles in enhancing plant growth and stress adaptation [27]. GA₃, a diterpenoid hormone, is known to play a role in promoting growth, seed germination and flowering, as well as improving drought tolerance by modulating the stomatolabelling and root structure [35, 67]. In drought conditions, GA₃ application has been shown to increase cell elongation and water uptake efficiency, thereby alleviating the inhibition of growth due to water scarcity [23, 63]

Similarly, IAA, the key hormone in auxin, regulates cell division, root development and growth of shoots and plays a key role in the response of plants to environmental stress. The use of IAA in leaf sprouts has been reported to increase root proliferation, increase nutrient uptake and stimulate antioxidant enzyme activity, which in turn contribute to improved drought tolerance [30]. In addition, IAA promotes osmotic adjustment and maintains cellular homeostasis, which enables plants to maintain their physiological functions under conditions of water stress [49]. The study suggests that the combined use of Metformin, GA₃ and IAA under different irrigation regimes will significantly increase the growth, yield and WU of peanuts. To test this, a two-year field trial was carried out to:i) evaluate the individual and pooled effects of Metformin, GA₃ and IAA on the growth and productivity of peanuts under various irrigation conditions. (ii) evaluate their impact on the chlorophyll content, nutrient assimilation and drought-resilience mechanisms of peanuts. By combining new and traditional biological stimulants, this research aims to provide a sustainable strategy for peanut production in water-limited environments, addressing a key challenge in modern farming.

## Methods

### Experimental site and environmental conditions

A two-year field experiment was conducted at the Ismailia Research Station, Agricultural Research Center, Ismailia Governorate, Egypt (latitude 30° 36'N, longitude 32° 17'E, altitude 1.0 m above sea level). The region is characterized by an arid climate, with mean annual precipitation of less than 30 mm. Seasonal temperatures range from 7 °C in winter to 40 °C in summer, while relative humidity varies between 43 and 60%. Climatic data, including daily air temperature, relative humidity, and solar radiation, were continuously recorded using an automatic weather station installed near the experimental field during both growing seasons (2022–2023). These data provided essential insights into the prevailing environmental conditions affecting crop growth and stress responses.

### Soil sample analysis

Before cultivation, soil samples were taken randomly from the experimental field area to determine physical and chemical soil parameters, as indicated in Table 1. All analysis methods are conventional and reported by Estefan et al. [26].

**Table 1:** Chemical and physical characteristics of initial soil sample before cultivation

Soil characteristics	Value	Soil characteristics	Value
Particle size distribution%:		Soluble cations (soil paste, mmole l^−1^):	
Sand	86.2	Ca^+2^	0.92
Silt	9.60	Mg^+2^	0.61
Clay	4.20	Na^+^	0.85
Textural Class	Sandy^a^	K^+^	0.19
Soil chemical properties:		Soluble anions (soil paste, mmole l^−1^):	
pH (1:2.5 soil suspension)	7.95	CO_3_^−2^	0.00
CaCO_3_%	1.42	HCO^−3^	1.36
Organic matter %	0.30	Cl^−^	0.70
EC_e_ (dS m^−1^, soil paste extract)	0.27	SO_4_^−2^	0.45
Soil physical properties:		Total macro- and micronutrients mg kg^−1^	
Bulk density g cm^−3^	1.52	N	46.34
SAR	5.90	P	18.56
ESP	4.50	K	349.1
SP	15.3	Fe	41.2
CEC	13.2	Mn	2.43
Moisture content %:		Zn	2.31
Field capacity	13.26		
Wilting point	5.22		
Available water	8.05		

^a^Using USAD Soil Texture Triangle, after [8]

### Soil preparation and fertilizer management in the experimental site

All peanut plots underwent double plowing in perpendicular directions during soil preparation for planting. Fertilization included the application of single superphosphate (12.5% P₂O₅) at a rate of 30 kg P₂O₅ per acre. Nitrogen was supplied using ammonium sulfate (20.6% N), applied in two equal doses one and two months after planting. Potassium was provided as potassium sulfate (48% K₂O) at a rate of 40 kg N per acre as a basal dose, along with an additional 36 kg K₂O per acre. Aydinsakir et al. [4] and Sezen et al. [66] have demonstrated that similar fertilization regimes optimize peanut growth, yield, and nutrient uptake under varying irrigation levels. These doses ensure adequate nutrient availability without excessive application that could lead to environmental loss or inefficiency. Apart from irrigation, all agronomic practices adhered to standard procedures for peanut cultivation in the region, following Egypt's Ministry of Agriculture guidelines.

### Plant material

The peanut cultivar"Giza 6"was used in this study. This cultivar is widely grown in Egypt due to its high yield potential, drought tolerance, and good seed quality. Prior to sowing, seeds were inoculated with Azotobacter bacteria to enhance biological nitrogen fixation and promote root development. Seeds were hand-sown on May 20 in both 2022 and 2023 at a spacing of 30 cm between plants and 60 cm between rows. Two weeks after emergence, seedlings were thinned to one per hill to minimize competition​ [39, 40]. Approximately two weeks post-sowing, seedlings were thinned to one per hill to reduce inter-plant competition.

### Experimental design and treatments

Under soil water stress conditions, the experiments were conducted over two consecutive summer seasons, 2022 and 2023. A split-split plot design with three replications was employed. Main plot treatments included three irrigation levels: full irrigation (100% of the recommended rate, at 2812.5 m^3^ ha⁻^1^, as the control), 80% irrigation (2250 m^3^ ha⁻^1^), and 60% irrigation (1687.5 m^3^ ha⁻^1^. Water was supplied using inline drip emitters with a flow rate of 4 L h⁻^1^ per emitter. Emitters were spaced 30 cm apart along lateral drip lines, which were 75 cm apart. In addition, irrigation scheduling was based on soil moisture monitoring, with water applied three times per week to maintain adequate root zone moisture.

Subplot treatments involved foliar application of plant growth stimulants with four treatment types: control, Metformin, gibberellin, and indole acetic acid. The sub-subplot treatments further varied the application rate of each stimulant (control, 5.0, 7.5, and 10.0 mg L⁻^1^). The experimental setup comprised 144 plots, each with an area of 10.5 m^2^ (3 × 3.5 m), containing three rows of plants cultivated on both sides of each row. The spacing between rows was set at 60 cm, with 30 cm between individual plants.

### Formula of the Metformin, gibberellin, and indole acetic acid

In this study, three plant growth stimulants were used as foliar bio-stimulants to enhance peanut resilience under drought stress conditions. Metformin hydrochloride (C₄H₁₁N₅·HCl, molecular weight: 165.62 g mol⁻1) was synthesized in the laboratory through a controlled chemical reaction involving dicyandiamide and dimethylamine, ensuring high purity (≥ 98%) for experimental application. The selection of Metformin as a plant bio-stimulant was based on its reported ability to regulate reactive oxygen species (ROS) homeostasis, enhance chlorophyll biosynthesis, and improve water use efficiency (WUE) in plants exposed to abiotic stress [22].

Gibberellic acid (GA₃, C₁₉H₂O₆, molecular weight 346.38 g mol⁻) was obtained from Sigma-Aldrich (USA) in the form of commercial grade plant growth regulators (purity < 90 percent) [13]. GA₃ plays a key role in stimulating cell elongation, seedling and root development [68]. GA₃ is known to increase stomatal conductivity and root water uptake under drought conditions, improving water-water contact and overall physiological performance of plants [16, 41].

Indole-3-acetic acid (IAA, C₁₀H₉NO₂, molecular weight: 175.19 g mol⁻1) was purchased from Sigma-Aldrich (USA) with a purity of ≥ 95%. IAA, as a natural auxin, is vital for root initiation, shoot elongation, and nutrient uptake [44]. Additionally, it contributes to osmotic adjustment and cellular homeostasis, thereby increasing the plant’s ability to tolerate drought-induced stress [51].

Wang et al., (2024) Metformin regulates cellulase production in Trichoderma reesei via calcium signaling and mitochondrial function. Microbial Cell Factories 23(1):314. https://doi.org/10.1186/s12934-024-02593-w

All growth stimulants were dissolved in deionized water before foliar application. To optimize their stability and absorption, the solutions were adjusted to a pH range of 6.5–7.0 using a 0.01 M phosphate buffer. The foliar treatments were applied using a hand-held sprayer, ensuring uniform coverage over the leaf surface. To prevent photodegradation and maximize uptake, applications were conducted early in the morning under low sunlight intensity.

### Preparation of metformin hydrochloride from dicyanodiamide

To a solution of 20 g of dicyandiamide, 100 mL of ethanol was added sequentially. After complete dissolution, 28 g of an aqueous dimethylamine solution (prepared by mixing dimethylamine and deionized water at a weight ratio of 40:100) was introduced dropwise over one hour in an ice bath. Upon completion of the addition, the reaction mixture was allowed to reach room temperature and stirred for an additional 8 h. Subsequently, dilute hydrochloric acid was added dropwise until the pH reached 2. Diethyl ether (150 mL) was added, and the mixture was left to separate by layering. The organic phase was removed, and the aqueous phase was evaporated to dryness. The residue was dissolved in a 1:9 mixture of isobutyl alcohol and ethanol, heated to 50 °C until fully dissolved, and then cooled on ice for 10 h to precipitate crystals. After filtration and drying, metformin hydrochloride was obtained with a yield of 98.2%.

### Data collection

#### Plant growth and yields

The harvesting process was conducted when peanut plants reached full physiological maturity, as indicated by yellowing of leaves and the development of hardened pods. For both growing seasons (2022 and 2023), harvest occurred in late September, approximately four months after sowing (May 20th). After harvesting, plant samples were taken randomly from the two inner slopes of each plot and carefully washed with distilled water to remove any residuals from the soil. The height of the plant was measured from 2 cm above the soil surface to the top of the main stem of the plant. Additional yield parameters, such as number of branches per plant, weight of seeds per plant, biomass yield in fresh and dried form and total seed yield, were recorded to assess the effects of the treatment.

### Water use efficiency

Water use efficiency (WUE) was evaluated to assess how effectively peanut plants utilized available water under different irrigation treatments based on the equation of Viets [76]. WUE, expressed as kg seed yield per cubic meter of water consumed (kg m⁻^3^), was calculated using the formula:

$$\text{WUE }= {\text{Seed yield in kg acre}}^{-1}/ {\text{actual consumptive used in }{\text{m}}^{3}\text{ acre}}^{-1}$$

#### Mineral composition

Plant material samples were oven-dried at 70 °C for 72 h, finely ground, and analyzed to determine peanut nutrient content on a dry weight basis. For nutrient analysis, the dried plant samples were wet-digested using a mixture of H₂SO₄ and HClO₄ (1:1 v/v) to quantify macronutrients (N, P, and K) and micronutrients (Fe, Zn, and Mn) following the method outlined by Piper [60]. Total nitrogen content was assessed using the Kjeldahl method [34]. Phosphorus levels were determined colorimetrically [56], and potassium content was measured using a flame photometer [54]. Additionally, chlorophyll and carotenoid levels in fresh leaves were quantified following the method of Sumanta et al. [72].

#### Proximate analysis

Crude protein content was calculated by multiplying the total nitrogen content of the seeds by a factor of 6.25, as described by Deyoe and Shellenberger [20]. The oil content of the seeds was determined using a Soxhlet apparatus with petroleum ether as the solvent, following the method outlined by AOAC [2]. The iodine value of the peanut oils was assessed using the equation provided by Hashim et al. [33] and Chowdhury et al. [14].

$$Iodine\;No.\;=\left(a-b\right)\times N\times126.9\times100/W\times100$$

Where a is the volume of sodium thiosulphate as blank, b is the sodium thiosulphate required for the sample, N is the normality, and w is the weight of the oil sample in grams. Oleic acid was determined according to Zhang et al. [82]. Finally, data was subjected to calculate the net return benefit for the peanut experiment at all treatments under study.

#### Statistical analysis

All data were analyzed statistically according to the experimental design using analysis of variance (ANOVA). The least significant difference (LSD) at 0.05 was used to determine significant differences among treatments. The statistical analysis was conducted using MSTAT-C software, version 2.1 (Michigan State University, USA, 1989 edition) for ANOVA and LSD calculations [31].

## Results

### Impact of Water Irrigation Rates and plant growth regulator on peanut growth and yield under soil water stress

The results presented in Table 2 highlight the effects of plant growth regulators and varying water irrigation rates on several key aspects of plant growth and yield, including plant height, number of branches, and fresh and dry weight. The data indicate that reducing irrigation from 100 to 60% decreases various morphological traits, such as plant height, branch number per plant, and fresh and dry shoot weight. Among the plant growth stimulants tested, Metformin was found to be the most effective treatment, outperforming the foliar applications of gibberellin (GA3) and indole-3-acetic acid (IAA), as well as the control treatment, in terms of plant height, branch number, and both fresh and dry weight. Among the plant growth stimulants tested, Metformin at 7.5 mg L⁻⁹ under 80 percent irrigation significantly increased the height of the plants (76.9 cm), the number of branches (17.7 per plant), the dry biomass (329.1 kg of biomass) and the total yield of the seeds (1,929.5 kg of seed). The interaction of irrigation level × stimulant type × concentration (S × C × I) was significant in the content of seed oil (LSD = 0.63), of crude protein (LSD = 0.83), and of iodine (LSD = 1.03), which demonstrated the statistically significant effect of the combination of the two treatments.

**Table 2:** Peanut growth and yield are affected by the foliar application of plant growth stimulants at different concentrations grown under different water irrigation

Water irrigation, % (I)	Plant growth stimulant sources (S)																					
	**Metformin**								**Gibberellin (GA**_**3**_**)**							**Indol acetic acid (IAA)**						
	**Plant growth stimulant concentration, mg l**^**−1**^** (C)**																					
	**Cont**		**5.0**	**7.5**		**10.0**	**Mean**		**Cont**	**5.0**		**7.5**	**10.0**		**Mean**	**Cont**		**5.0**	**7.5**		**10.0**	**Mean**
Plant height, cm																						
100	55.7		69.8	83.5		78.6	71.9		54.9	62.5		75.3	88.9		70.4	55.2		65.8	72.6		80.8	68.6
80	53.6		64.2	76.9		72.2	66.7		54.1	57.9		68.7	76.2		64.2	53.8		61.6	67.1		74.5	64.3
60	50.9		53.7	65.8		61.4	57.9		51.4	54.7		62.1	71.8		60.0	50.7		54.7	63.9		69.2	59.6
Mean	53.4		62.6	75.4		70.7	65.5		53.5	58.4		68.7	78.9		64.9	53.2		60.7	67.9		74.8	64.2
No. of branches, plant^−1^																						
100	11.4		15.5	18.9		17.3	15.8		10.9	13.7		15.5	17.7		14.5	12.0		14.8	17.6		18.2	15.7
80	11.1		14.2	17.7		15.9	14.7		11.3	13.4		14.9	16.9		14.1	10.8		13.7	14.9		16.5	14.0
60	9.5		10.7	14.8		13.8	12.2		8.9	10.8		11.7	13.6		11.3	10.1		11.3	13.1		14.1	12.2
Mean	10.7		13.5	17.1		15.7	14.2		10.4	12.6		14.0	16.1		13.3	10.9		13.3	15.2		16.3	13.9
Fresh weight, kg acre^−1^																						
100	1589.4		1939.2	2844.1		2561.6	2233.6		1548.3	1780.5		2175.6	2682.1		2046.6	1578.9		1874.6	2382.7		2748.3	2146.1
80	1522.9		1795.6	2928.5		2780.2	2256.8		1537.6	1637.9		1940.2	2338.5		1863.6	1528.5		1755.1	2168.3		2623.8	2018.9
60	1451.7		1628.5	2573.9		2368.9	2005.8		1446.2	1519.3		1755.7	1962.3		1670.9	1447.9		1582.7	1894.9		2157.5	1770.8
Mean	1521.3		1787.8	2782.2		2570.2	2165.4		1510.7	1645.9		1957.2	2327.6		1860.4	1518.4		1737.5	2148.6		2509.9	1978.6
Dry weight, kg acre^−1^																						
100	165.9		189.2	276.9		253.7	221.4		169.3	176.9		245.7	267.9		214.9	164.9		188.6	211.4		289.1	213.5
80	157.2		213.8	329.1		291.4	247.9		153.8	157.6		193.3	239.6		186.1	155.7		167.3	185.8		252.6	190.4
60	148.5		164.3	227.6		205.8	186.6		142.9	149.8		172.5	208.1		168.3	146.5		159.2	172.6		225.2	175.9
Mean	157.2		189.1	277.9		250.3	218.6		155.3	161.4		203.8	238.5		189.9	155.7		171.7	189.9		255.6	193.2
LSD at 0.05																						
Parameter		S			C			I			S × C			S × I			C × I			S × C × I		
Plant height		1.8			2.6			2.0			2.3			1.9			2.2			3.2		
No. of branches		1.1			1.3			0.9			1.0			1.4			1.3			1.3		
Fresh weight		76.2			44.9			65.4			70.3			64.9			81.0			69.0		
Dry weight		7.9			5.3			3.1			4.8			3.8			2.2			4.6		

*S *Plant growth stimulant sources, *C *Plant growth stimulant concentration and *I *Water irrigation

### Impact of water irrigation rates and plant growth regulator on total yield and yield content of peanut seeds

The data presented in Table 3 demonstrate that the highest 100-seed weight (83.8 g) and total yield (1612.5 kg per acre) were obtained under the 100% irrigation rate combined with a 7.5 mg L⁻^1^ concentration of the plant growth stimulant metformin. It was observed that reducing the irrigation rate from 100 to 60% resulted in a 32%, 27%, and 28% decrease in total peanut yield when Metformin, GA3, and IAA were applied, respectively. The optimal treatment for both 100-seed weight and yield was the combination of 100% irrigation with a foliar application of Metformin at a concentration of 7.5 mg L⁻^1^, followed by a foliar application of IAA at 10 mg L⁻^1^ under the same irrigation conditions.

**Table 3:** Total yield of peanut plants affected by foliar application of plant growth stimulant at different concentration grown under different water irrigation

Water irrigation, % (I)	Plant growth stimulant sources (S)																					
	**Metformin**								**Gibberellin (GA**_**3**_**)**							**Indol acetic acid (IAA)**						
	**Plant growth stimulant concentration, mg l**^**−1**^** (C)**																					
	**Cont**	**5.0**		**7.5**		**10.0**	**Mean**		**Cont**	**5.0**		**7.5**	**10.0**		**Mean**	**Cont**		**5.0**	**7.5**		**10.0**	**Mean**
Weight of 100 seeds, gm																						
100	56.9	78.2		83.8		80.5	74.9		57.2	66.5		73.8	79.3		69.2	56.5		67.7	76.2		80.9	70.3
80	53.0	75.7		81.6		78.3	72.2		53.7	62.9		67.5	74.9		64.8	54.3		65.3	71.9		78.5	67.5
60	49.5	63.4		74.9		69.9	64.4		48.4	57.1		62.9	68.1		59.1	49.7		59.6	68.5		73.8	62.9
Mean	53.1	72.4		80.1		76.2	70.5		53.1	62.2		68.1	74.1		64.4	53.5		64.2	72.2		77.7	66.9
Total yield, kg acre^−1^																						
100	983.2	1279.8		1612.5		1547.7	1355.8		971.5	1153.1		1369.8	1474.3		1242.2	979.8		1230.9	1426.4		1598.7	1308.9
80	861.7	1145.3		1593.9		1370.2	1242.8		844.8	986.9		1171.5	1298.6		1075.5	853.5		995.3	1268.8		1329.4	1111.8
60	759.3	912.9		1088.5		1142.8	975.9		763.6	839.4		927.9	1089.1		905.0	757.3		879.6	974.5		1153.9	941.3
Mean	868.1	1112. 7		1431.6		1353.6	1191.4		859.9	993.1		1156.4	1287.3		1074.2	863.5		1035.3	1223.2		1360.7	1120.7
LSD at 0.05																						
Parameter			S		C			I			S × C			S × I			C × I			S × C × I		
Weight of 100 seeds			1.1		0.9			1.0			1.2			0.8			0.5			0.6		
Total yield			98.9		112.9			111.5			88.9			90.3			76.2			66.4		

*S *Plant growth stimulant sources, *C *Plant growth stimulant concentration and *I *Water irrigation

### Impact of water irrigation rates and plant growth regulator on Water Use Efficiency (WUE)

The sources and concentrations of plant growth regulators and irrigation levels significantly affected water use efficiency (WUE) (Table 4). The trends in Table 4 align with the previously discussed patterns for all plant parameters. The highest WUE values (1.92 and 1.82 kg m⁻^3^) were recorded at an irrigation level of 60%, in combination with IAA and GA at a concentration of 10 mg L⁻^1^, representing increases of 54.7% and 52.7%, respectively, compared to the control treatment. At an irrigation level of 80%, Metformin at a concentration of 7.5 mg L⁻^1^ resulted in a 50.8% increase in WUE compared to the control treatment.

**Table 4:** Water use efficiency of peanut plants affected by foliar application of plant growth stimulant at different concentration grown under different water irrigation

Water irrigation, % (I)	Plant growth stimulant sources (S)											
	**Metformin**				**Gibberellin (GA**_**3**_**)**				**Indol acetic acid (IAA)**			
	**Plant growth stimulant concentration, mg l**^**−1**^** (C)**											
	**Cont**	**5.0**	**7.5**	**10.0**	**Cont**	**5.0**	**7.5**	**10.0**	**Cont**	**5.0**	**7.5**	**10.0**
	**Water use efficiency, kg m**^**−3**^											
**100**	0.87b	1.14b	1.43b	1.38c	0.86b	1.03b	1.22b	1.31b	0.87b	1.09b	1.27c	1.42b
**80**	0.96b	1.27b	1.77a	1.52b	0.94b	1.10b	1.30b	1.44b	0.95b	1.11b	1.41b	1.48b
**60**	1.27a	1.52a	1.61b	1.71a	1.27a	1.40a	1.55a	1.82a	1.26a	1.47a	1.62a	1.92a
**Mean**	1.033	1.310	1.603	1.537	1.023	1.177	1.357	1.523	1.027	1.223	1.433	1.607
**LSD at 0.05**	0.09	0.13	0.11	0.15	0.24	0.13	0.20	0.13	0.21	0.15	0.13	0.2

*S* Plant growth stimulant sources, *C* Plant growth stimulant concentration and *I* Water irrigation

### Impact of water irrigation rates and plant growth regulator on Pigment Content of peanut plant leaves

The results presented in Table 5 indicate that the treatment with Metformin at a concentration of 7.5 mg L⁻^1^, followed by IAA at 10 mg L⁻^1^, was the most effective in enhancing the total chlorophyll and carotenoid content. Specifically, combining 100% water irrigation with foliar application of Metformin at 7.5 mg L⁻^1^ resulted in increases of 48% and 44.6% in leaf pigment content compared to the control treatments. Furthermore, Table 5 demonstrates that a water regime at 60% of the peanut plant's actual irrigation requirement reduced total chlorophyll and carotenoid content. This decrease in pigment levels is attributed to water deficiency, which negatively impacts various physiological and biological processes, including plant growth, flowering, fruit development, stomatal closure, and reduced transpiration.

**Table 5:** Leaves pigment contents of peanut plants affected by foliar application of plant growth stimulant at different concentration grown under different water irrigation

Water irrigation, % (I)	Plant growth stimulant sources (S)																					
	**Metformin**								**Gibberellin (GA**_**3**_**)**							**Indol acetic acid (IAA)**						
	**Plant growth stimulant concentration, mg l**^**−1**^** (C)**																					
	**Cont**		**5.0**	**7.5**		**10.0**	**Mean**		**Cont**	**5.0**		**7.5**	**10.0**		**Mean**	**Cont**		**5.0**	**7.5**		**10.0**	**Mean**
Total chlorophyll, mg g^−1^ FW																						
100	0.66		0.78	1.27		1.11	0.95		0.68	0.71		0.86	1.07		0.83	0.67		0.73	0.92		1.13	0.86
80	0.51		0.69	0.98		0.86	0.76		0.54	0.63		0.75	0.82		0.69	0.52		0.65	0.80		0.93	0.73
60	0.47		0.57	0.77		0.58	0.60		0.49	0.52		0.68	0.74		0.61	0.46		0.54	0.71		0.85	0.64
Mean	0.55		0.68	1.01		0.85	0.77		0.57	0.62		0.76	0.88		0.71	0.55		0.64	0.81		0.97	0.74
Carotenoid content, mg g^−1^ FW																						
100	1.59		1.97	2.87		2.64	2.27		1.57	1.76		1.92	2.53		1.95	1.61		1.82	2.33		2.68	2.11
80	1.54		1.72	2.54		2.19	1.99		1.51	1.68		1.86	2.06		1.78	1.55		1.76	1.87		2.15	1.83
60	1.47		1.58	2.11		1.93	1.77		1.44	1.59		1.74	1.93		1.68	1.45		1.64	1.72		2.03	1.71
Mean	1.53		1.76	2.51		2.25	2.01		1.51	1.68		1.84	2.17		1.79	1.54		1.74	1.97		2.29	1.88
	LSD at 0.05																					
Parameter		S			C			I			S × C			S × I			C × I			S × C × I		
T. chlorophyll		0.21			0.11			0.09			0.13			0.14			0.13			0.12		
Carotenoids		0.27			0.15			0.17			0.26			0.22			0.27			0.17		

*S* Plant growth stimulant sources, *C *Plant growth stimulant concentration and *I *Water irrigation

### Impact of Water Irrigation Rates and plant growth regulator on macronutrient contents of peanut seeds

The data presented in Table 6 demonstrate that irrigation at 100% of the water requirement for peanut plants resulted in the optimal macronutrient content. In contrast, a reduction in irrigation to 60% of the required water level led to the lowest macronutrient content, likely due to the detrimental effects of water scarcity on plant growth and yield. However, foliar application of Metformin at a concentration of 7.5 mg L⁻^1^, combined with irrigation at 80% of the water requirement, resulted in the highest nitrogen content in peanut seeds. Moreover, the data indicate that applying plant growth stimulants at varying concentrations had differing impacts on macronutrient content. Metformin at 7.5 mg L⁻^1^ was the most effective, followed by IAA at 10.0 mg L⁻^1^. Metformin's role in enhancing melatonin production, as noted by Li et al. [47], plays a key part in plant stress tolerance. Melatonin (MT), as a biological regulator, has been shown to improve stress resistance by increasing chlorophyll content, enhancing photosynthetic carbon uptake, activating the ROS scavenging system, and delaying leaf senescence.

**Table 6:** Macronutrients in peanut plants are affected by foliar application of plant growth stimulant at different concentrations grown under different water irrigation

Water irrigation, % (I)	Plant growth stimulant sources (S)																				
	**Metformin**							**Gibberellin (GA**_**3**_**)**							**Indol acetic acid (IAA)**						
	**Plant growth stimulant concentration, mg l**^**−1**^** (C)**																				
	**Cont**	**5.0**	**7.5**		**10.0**	**Mean**		**Cont**	**5.0**		**7.5**	**10.0**		**Mean**	**Cont**		**5.0**	**7.5**		**10.0**	**Mean**
Nitrogen content, %																					
100	2.27	3.49	5.63		5.16	4.14		2.29	3.31		4.87	5.25		3.93	2.29		3.37	4.52		5.39	3.89
80	1.94	2.43	5.91		5.32	3.90		1.88	3.06		3.49	4.13		3.14	1.91		2.82	3.74		4.35	3.21
60	1.77	2.09	5.49		4.93	3.57		1.76	1.88		2.04	2.39		2.02	1.73		2.16	2.38		3.18	2.36
Mean	1.99	2.67	5.68		5.14	3.87		1.98	2.75		3.47	3.92		3.03	1.98		2.78	3.55		4.31	3.15
Phosphorus content, %																					
100	1.18	1.73	2.82		2.36	2.02		1.18	1.47		1.88	1.97		1.63	1.16		1.34	1.94		2.08	1.63
80	1.12	1.39	2.37		1.72	1.65		1.11	1.29		1.47	1.86		1.43	1.13		1.21	1.58		1.83	1.44
60	0.81	1.18	1.92		1.54	1.36		0.85	1.15		1.29	1.51		1.20	0.83		1.16	1.39		1.69	1.27
Mean	1.04	1.43	2.37		1.87	1.68		1.05	1.30		1.55	1.78		1.42	1.04		1.24	1.64		1.87	1.45
Potassium content, %																					
100	1.94	3.35	5.49		4.41	3.80		1.89	2.86		3.75	4.89		3.35	1.91		2.75	3.93		4.71	3.33
80	1.50	2.72	4.43		3.96	3.15		1.53	2.54		2.82	3.36		2.56	1.48		2.42	3.56		3.95	2.85
60	1.21	2.22	3.88		3.53	2.71		1.21	2.20		2.34	2.87		2.16	1.19		1.98	2.31		3.53	2.25
Mean	1.55	2.76	4.60		3.97	3.22		1.54	2.53		2.97	3.71		2.69	1.53		2.38	3.27		4.06	2.81
	LSD at 0.05																				
Parameter	S			C			I			S × C			S × I			C × I			S × C × I		
Nitrogen	0.54			0.23			0.33			0.18			0.21			0.19			0.21		
Phosphorus	0.12			0.11			0.14			0.11			0.15			0.13			0.12		
Potassium	0.52			0.89			0.51			0.44			0.33			0.23			0.35		

*S* Plant growth stimulant sources, *C* Plant growth stimulant concentration and *I* Water irrigation

### Impact of irrigation rates and plant growth regulators on micronutrient content of peanut seeds

The results in Table 7 reveal that the trends for micronutrient content in peanut seeds mirrored those of macronutrient content, with the lowest concentrations of Fe, Mn, and Zn observed under 60% irrigation. Regarding plant growth stimulants, the data indicate that foliar application of Metformin at 7.5 mg L⁻^1^ was the most effective treatment, followed by the application of gibberellin and IAA at 10.0 mg L⁻^1^, which showed superior results across all examined micronutrient levels. The optimal treatment was the combination of foliar Metformin at 7.5 mg L⁻^1^ and irrigation at 80% of the water requirement for peanut plants. Table 7 further illustrates that Metformin was the most effective plant growth stimulant in alleviating peanut drought stress [37], with IAA and GA also demonstrating positive effects. Additionally, Metformin enhanced the uptake of micronutrients by peanut plants, further supporting its efficacy in improving overall plant nutrient status.

**Table 7:** Micronutrient content in peanut plants affected by foliar application of plant growth stimulant at different concentration grown under different water irrigation

Water irrigation, % (I)	Plant growth stimulant sources (S)																				
	**Metformin**							**Gibberellin (GA**_**3**_**)**							**Indol acetic acid (IAA)**						
	**Plant growth stimulant concentration, mg l**^**−1**^** (C)**																				
	**Cont**	**5.0**	**7.5**		**10.0**	**Mean**		**Cont**	**5.0**		**7.5**	**10.0**		**Mean**	**Cont**		**5.0**	**7.5**		**10.0**	**Mean**
Iron content, mg kg^−1^																					
100	102.3	253.7	355.1		293.2	251.1		99.5	229.3		329.9	275.8		233.6	101.8		215.9	308.3		259.5	221.4
80	97.5	237.3	327.5		281.6	235.9		95.1	215.6		310.1	256.2		219.3	93.4		207.2	288.4		239.3	207.1
60	94.9	198.9	295.7		278.8	217.1		91.7	187.3		281.3	265.2		206.4	90.8		169.8	272.9		246.9	195.1
Mean	98.2	229.9	326.1		284.5	234.7		95.4	210.7		307.1	265.7		219.8	95.3		197.63	289.9		248.6	207.9
Manganese content, mg kg^−1^																					
100	26.7	41.3	66.5		59.6	48.5		24.1	38.7		61.3	53.9		44.5	27.3		36.5	59.7		50.1	43.4
80	22.8	39.6	54.8		50.2	41.9		21.6	33.1		51.2	46.6		38.1	22.4		31.5	48.9		39.8	35.7
60	18.6	35.3	49.6		47.8	37.8		17.5	31.8		45.3	41.5		34.0	17.7		29.5	41.9		38.7	31.9
Mean	22.7	38.7	56.9		52.5	42.7		21.1	34.5		52.6	47.3		38.9	22.5		32.5	50.2		42.9	37.0
Zinc content, mg kg^−1^																					
100	131.9	258.2	413.5		394.3	299.5		128.5	233.9		379.2	392.5		283.5	123.8		247.5	352.6		322.7	261.7
80	117.2	231.7	413.5		394.3	289.2		109.7	216.2		346.9	372.1		261.2	116.2		205.4	352.6		322.7	249.2
60	102.4	215.8	392.7		376.1	271.8		99.8	196.4		355.1	338.6		247.5	100.9		180.6	329.6		308.5	229.9
Mean	117.2	235.2	406.6		388.2	286.8		112.7	215.5		360.4	367.7		264.1	113.6		211.27	344.9		317.9	246.9
	LSD at 0.05																				
Parameter	S			C			I			S × C			S × I			C × I			S × C × I		
Iron	10.6			8.9			4.3			8.1			5.2			4.4			6.1		
Manganese	0.5			0.3			0.7			0.6			0.4			0.2			0.5		
Zinc	9.7			7.8			8.9			7.2			5.8			9.1			7.3		

*S* Plant growth stimulant sources, *C* Plant growth stimulant concentration and *I* Water irrigation

According to Feng et al. [28], Metformin can enhance the production of reactive oxygen species (ROS) through reverse electron flow into mitochondrial complex I. It also aids in repairing the damaged tri-carboxylic acid (TCA) cycle and stabilizing mitochondrial function.

### Impact of water irrigation rates and plant growth regulator on Peanut seed quality parameters

The results presented in Table 8 demonstrate that the foliar application of plant growth stimulants enhances seed oil content, iodine number, oleic acid content, and crude protein levels. The response to foliar applications of plant growth stimulants (Metformin, GA, and IAA) varied depending on the specific stimulant used. According to the data in Table 8, the foliar application of Metformin at 7.5 mg L⁻1 proved to be the most effective treatment compared to the other treatments studied. Additionally, the interaction treatment combining 80% water irrigation with the foliar application of Metformin at 7.5 mg L⁻1 emerged as the most effective in improving seed oil content, iodine number, oleic acid content, and crude protein in peanut plants. This treatment outperformed the foliar application of indole acetic acid at 10 mg L⁻1 under the same 80% water irrigation conditions.

**Table 8:** Seed oil content, iodine no. and oleic acid content in peanut plants affected by foliar application of plant growth stimulant at different concentration grown under different water irrigation

Water irrigation, % (I)	Plant growth stimulant sources (S)																				
	**Metformin**							**Gibberellin (GA**_**3**_**)**							**Indol acetic acid (IAA)**						
	**Plant growth stimulant concentration, mg l**^**−1**^** (C)**																				
	**Cont**	**5.0**	**7.5**		**10.0**	**Mean**		**Cont**	**5.0**		**7.5**	**10.0**		**Mean**	**Cont**		**5.0**	**7.5**		**10.0**	**Mean**
Seed oil content, %																					
100	20.56	26.88	32.74		28.59	27.19		20.41	23.55		26.80	29.52		25.07	20.62		24.75	27.39		29.71	25.62
80	18.73	25.17	36.96		30.28	27.79		18.70	21.83		25.65	31.58		24.44	18.58		22.49	26.73		31.36	24.79
60	16.88	24.36	29.24		25.72	24.05		16.93	19.26		22.79	27.67		21.66	17.11		20.97	23.68		28.72	22.62
Mean	18.72	25.47	32.98		28.20	26.34		18.68	21.55		25.08	29.59		23.72	18.77		22.74	25.93		29.93	24.34
Iodine number, mg 100 kg^−1^																					
100	55.64	64.91	76.29		70.47	66.83		55.26	62.77		69.28	73.45		65.19	55.40		63.35	71.48		74.71	66.24
80	53.50	62.74	80.38		72.73	67.34		53.38	60.51		67.65	75.70		64.31	53.82		61.70	73.57		76.19	66.32
60	48.78	57.88	74.67		64.41	61.44		49.07	54.70		59.81	66.26		57.46	48.95		55.29	63.85		69.30	59.35
Mean	52.64	61.84	77.11		69.20	65.20		52.57	59.33		65.58	71.80		62.32	52.72		60.11	69.63		73.40	63.97
Oleic acid content, mg 100 kg^−1^																					
100	35.17	52.83	81.59		75.39	61.25		34.92	48.83		67.72	72.12		55.89	35.10		50.75	69.58		76.84	58.07
80	33.85	56.71	84.92		77.45	63.23		33.76	52.58		69.24	78.85		58.61	33.89		54.23	73.93		79.38	60.36
60	30.41	49.89	78.66		70.80	57.44		30.25	45.76		52.89	64.55		48.36	30.57		47.82	66.59		68.92	53.48
Mean	33.14	53.14	81.72		74.55	60.64		32.98	49.06		63.28	71.84		54.29	33.19		50.93	70.03		75.05	57.30
Crude protein, %																					
100	14.19	21.81	35.19		32.25	25.86		14.31	20.69		30.44	32.81		24.56	14.31		21.06	28.25		33.69	24.33
80	12.13	15.19	36.94		33.25	24.38		11.75	19.12		21.81	25.81		19.62	11.94		17.63	23.38		27.20	20.04
60	11.06	13.06	34.31		30.81	22.31		11.00	11.75		12.75	14.94		12.61	10.81		13.50	14.88		19.88	14.77
Mean	12.46	16.69	35.48		32.10	24.18		12.35	17.19		21.67	24.52		18.93	12.35		17.39	22.17		26.92	19.71
	LSD at 0.05																				
Parameter	S			C			I			S × C			S × I			C × I			S × C × I		
Seed oil content	0.62			0.44			0.98			o.44			0.63			0.55			0.82		
Iodine no	1.20			0.99			0.67			0.49			0.83			0.67			0.49		
Oleic acid	1.32			1.21			1,54			1.22			1.08			1.86			1.01		
Crude protein	1.01			1.11			0.93			0.88			1.03			0.93			0.82		

*S* Plant growth stimulant sources, *C* Plant growth stimulant concentration and *I* Water irrigation

## Discussion

This study investigates how optimizing irrigation rates, along with the application of plant growth regulators (PGRs) and their respective concentrations, can enhance peanut crop development, yield, nutritional value, and water use efficiency from an ecophysiological perspective. Our findings demonstrate that using Metformin as a plant growth regulator positively influences peanut crops'growth, yield, total yield, and nutritional value across various irrigation levels.

The optimal irrigation rate is critical in determining peanut yield [4, 66]. Numerous studies have emphasized the significant impact of irrigation practices on peanut plant growth. For instance, it has been reported that plant height and biomass accumulation in peanuts increase progressively with higher irrigation rates. Schimpl et al. [64] outlined the mechanism of drought stress and its impact on the developmental characteristics of peanut plants, such as plant height, attributing this to the plant's strong resistance to turgor pressure, which supports proper cell expansion. Conversely, reducing irrigation from 100 to 60% leads to soil compaction, negatively affecting branch development [74]. Soil water stress has been shown to reduce dry matter accumulation in plants due to diminished photosynthesis due to insufficient stomatal function [46, 59]. Our study aligns with these observations, as similar patterns were reported in recent research [18, 21, 24, 25, 52, 77, 78, 83, 84].

Metformin acts as a modulator of reactive oxygen species (ROS), effectively mitigating oxidative stress by enhancing antioxidant enzyme activity and stabilizing mitochondrial function. This function is crucial in protecting cell membranes and organelles from oxidative damage induced by drought stress, ultimately enhancing cellular stability and longevity. By improving antioxidant enzyme activities such as superoxide dismutase and catalase, Metformin helps in reducing lipid peroxidation, which contributes to the overall resilience of peanut plants under limited water conditions. The positive effects observed in this study may be attributed to Metformin's high xylem mobility, which facilitates its rapid absorption and translocation within the plant. Previous studies have demonstrated that Metformin enhances plant drought tolerance by regulating electron transport in mitochondria and maintaining metabolic stability under stress conditions. effectively mitigating oxidative stress by enhancing antioxidant enzyme activity and stabilizing mitochondrial function. The positive effects observed in this study may be attributed to Metformin’s high xylem mobility, with a log Kow ranging from 1 to 2.5 [62]. More recently, it has been shown that even more water-soluble compounds (log Kow = − 1) exhibit substantial mobility in the xylem [19]. Additionally, foliar application of Metformin at a concentration of 7.5 mg L⁻1 was the most effective treatment for all growth parameters assessed in the peanut plants, followed by foliar application of IAA at 10 mg L⁻1. Zafar et al. [79] found that IAA treatment mitigated the stress-induced decline in metabolic characteristics and plant development in a dose-dependent manner.

Drought-induced yield reductions are commonly attributed to several factors, including a decrease in the rate of photosynthesis [29]. Zlatev and Lidon [85] reported that a high leaf area ratio provides a larger photosynthetic area under favorable growth conditions, thereby enhancing growth. Furthermore, studies on plant growth stimulants suggest that foliar application of Metformin in peanut plants outperforms GA3 and IAA in terms of both yield and yield components. Mahmud and Chong [50] highlighted that biofertilizers improve plant growth through direct and indirect mechanisms. Direct mechanisms include nitrogen fixation, phytohormone production, and phosphate solubilization, while indirect mechanisms protect plants from the harmful effects of pathogens. Our results corroborated these findings, with Metformin applied at 7.5 mg L⁻^1^ being the most effective treatment, followed by IAA at 10 mg L⁻^1^. These treatments positively influenced yield and 100-seed weight, likely due to exogenous IAA increasing endogenous IAA and GA3 levels in the leaves compared to the control, regardless of water conditions.

Metformin appears to mimic the action of natural nitrogen compounds, potentially enhancing the transport of Metformin across membranes, which may explain the unexpected allocation in oilseeds [22]. Additionally, exogenous IAA has been shown to alleviate stress-induced oxidative damage, growth inhibition, water deficit, and leaf senescence by maintaining higher chlorophyll content, optimizing antioxidant defenses, and reducing the expression of senescence marker genes (SAG101 and SAG102) in water-stressed leaves [48].

Water scarcity and environmental concerns present significant challenges, underscoring the need for sustainable peanut crop management strategies. Achieving optimal yields while conserving water resources and minimizing nutrient losses requires a balanced approach to irrigation. Traditional full irrigation methods have been shown to wastewater resources, resulting in low water use efficiency (WUE). Therefore, adopting drip irrigation is essential for enhancing the sustainability of peanut production. By implementing precise irrigation timing and optimizing foliar fertilizer application rates, farmers can improve water efficiency and mitigate potential environmental impacts [1].

WUE, a key indicator of the amount of peanuts produced per unit of water consumed, was used to evaluate peanut production under different irrigation treatments. Table 4 illustrates how varying irrigation regimes affect water use efficiency in peanut plants. Cantore et al. [11] reported that the impact of irrigation levels on WUE can vary depending on the extent of water stress during different growing seasons. Under conditions of low water stress, transpiration decreases more than photosynthesis due to slight stomatal closure, leading to increased WUE.

Table 5 highlights how different irrigation rates influence the pigment content of peanut leaves. Reducing the water supply to 60% of the required irrigation decreased leaf chlorophyll and carotenoid contents. This reduction in pigment content indicates water deficiency, which affects several physiological and biological processes essential for plant growth, including flowering, fruit development, and stomatal regulation, ultimately reducing transpiration. Lawlor [45] emphasized that manipulating water availability typically reduces leaf area, which lowers transpiration rates, allowing plants to utilize available water more efficiently. Reductions in other important biochemical markers, such as total soluble proteins, sugars, ascorbic acid, phenolic compounds, and yield-related attributes under water stress conditions, accompanied this decrease in pigment content.

Moreover, the application of various plant growth stimulants, such as Metformin, gibberellin, and indole acetic acid (IAA), showed promising results in mitigating the effects of water stress on peanut pigment content, as shown in Table 5. Among these stimulants, foliar application of Metformin was the most effective in alleviating the negative impacts of water stress on peanut pigment content, followed by IAA and gibberellin. Li et al. [48] explained that exogenous application of IAA led to a significant increase in endogenous IAA, gibberellin, abscisic acid (ABA), and polyamine (PAs) levels without affecting cytokinin content under water stress conditions. The increase in endogenous IAA levels supported polyamine metabolism by enhancing enzyme activity and increasing the transcript levels of polyamine-related genes, such as arginine decarboxylase, ornithine decarboxylase, and S-adenosylmethionine decarboxylase.

Changhai et al. [12] defined drought as an abiotic stressor caused by insufficient water availability to meet the plant's needs. Drought stress occurs when the rate of water intake by plants is significantly lower than the water lost through transpiration. Abiotic stresses, particularly drought, harm crop growth and yield, especially under field conditions [75]. Furthermore, the data presented in Table 6 indicate that foliar application of Metformin, GA3, and IAA positively influences peanut seeds'nitrogen, phosphorus, and potassium content. This suggests that the foliar application of these plant growth stimulants enhances the uptake and distribution of minerals and other dissolved substances from the soil to the plants. Li et al. [48] reported that the increased concentration of endogenous IAA resulting from IAA pretreatment significantly elevated the levels of endogenous IAA, GA3, and ABA under water stress conditions. Eggen and Lillo [22] proposed that organic cation transporters (OCTs) may play a crucial role in plant metformin accumulation compared to other eukaryotes. However, the contribution of other transport proteins remains unclear.

Drought is a complex stressor that affects plants at multiple levels of their biological organization [86]. Significant changes in water relations, biochemical and physiological processes, membrane integrity, and the ultrastructure of subcellular organelles characterize the dehydration process during drought. As shown in Table 8, reducing irrigation to 60% of the total water requirements for peanut plants adversely affects seed quality parameters. This reduction in the water regime from 100 to 60% led to decreased seed oil content, likely due to the plant's mechanisms for coping with water stress, which involves lowering carbohydrate levels in plant tissues [59]. In light of these findings, it is noteworthy that Metformin, identified as the most effective plant growth stimulant in this study, highlights the potential of using innovative technologies that involve specific classes of plant growth stimulants, such as Metformin. Metformin is a biguanide derivative that exhibits strong hydrophilic properties and exists as a monoprotonated cation. Kulkarni et al. [43] further emphasized that the beneficial effects of Metformin on energy metabolism and aging are partly attributed to its direct targeting of key energy sensors.

## Conclusion

This study provides compelling evidence that Metformin, a plant growth stimulant, significantly enhances peanut growth and yield under soil water stress conditions. The findings indicate that Metformin, particularly at a concentration of 7.5 mg L⁻^1^, improves essential growth parameters such as plant height, branch number, and biomass yield, thus increasing the overall productivity of peanut crops in water-limited environments. Furthermore, the study demonstrates that metformin application under an 80% irrigation level optimally enhances water use efficiency (WUE) and nutrient uptake, suggesting its viability as an eco-friendly and sustainable agricultural intervention. The combined use of drip irrigation and foliar application of Metformin is shown to mitigate the adverse effects of drought, conserve water, and maintain crop health, making it an effective strategy for sustainable agriculture. The results emphasize the potential of Metformin to improve resilience to abiotic stresses, thereby contributing to stable yields in regions affected by water scarcity. The efficacy of Metformin as a plant growth stimulant offers promising avenues for future research into its broader applications across various crop species, establishing a foundational approach to address the challenges of climate-induced water stress in agriculture.

## Data Availability

All data are included in manuscript.
